# Anterior Full‐endoscopic Single‐port Double Transcorporeal Spinal Cord Decompression for Noncontinuous Two‐segment Cervical Spondylotic Myelopathy: A Technical Note

**DOI:** 10.1111/os.13988

**Published:** 2024-01-29

**Authors:** Gang Chen, Zhijun Xin, Weijun Kong, Fujun Wu, Xuyan Li, Yanyu Qiao, Xiang Yan, Wenbo Liao

**Affiliations:** ^1^ Department of Orthopedics The Second Affiliated Hospital of Zunyi Medical University Zunyi China; ^2^ Department of Orthopedic Surgery The Affiliated Hospital of Zunyi Medical University Zunyi China

**Keywords:** Cervical spondylotic myelopathy, Full‐endoscopic operation, Minimally invasive spine surgery, Noncontiguous levels, Transcorporeal approach

## Abstract

**Objective:**

In clinical practice, noncontinuous two‐segment spinal cord cervical spondylosis is a particular form of cervical degenerative disease. Traditional anterior open surgery frequently comes with severe trauma, risks, and debatable treatment options. This study aimed to describe for the first time a novel minimally invasive technique, namely, anterior full‐endoscopic single‐port double transcorporeal spinal cord decompression for the treatment of patients with noncontinuous two‐segment cervical spondylotic myelopathy.

**Method:**

From February 2020 to May 2021, five patients with noncontinuous two‐segment cervical spondylotic myelopathy were treated with anterior full‐endoscopic single‐port double transcorporeal spinal cord decompression. Two bone channels were established by the trephine through the vertebral body oblique upward and downward to the herniated disc osteophyte complex, and the full‐endoscopic system could decompress the spinal cord through the channels. All cases were followed up for over 2 years. The modified Japanese Orthopaedic Association (mJOA) score and visual analogue scale (VAS) score before and after operation and during follow‐up were used to evaluate the clinical effectiveness. Radiological examinations, including CT and MRI, were utilized to evaluate the efficacy of spinal cord decompression and bone channel repair.

**Results:**

All operations were successfully completed and the average operation time was 185 min, with no operation‐related complications. Compared with the preoperative evaluation, the mJOA score and VAS score were improved at each time point after operation and follow‐up. Postoperative CT and MRI scans showed that the intervertebral disc‐osteophyte complex was removed through the vertebral bone passage, and the spinal cord was fully decompressed. After 24 months of follow‐up, CT and MRI scans showed that the bone channel was almost repaired and healed.

**Conclusion:**

Anterior full‐endoscopic single‐port double transcorporeal spinal cord decompression is an effective minimally invasive technique for noncontinuous two‐segment cervical spondylosis. It provides precise and satisfactory spinal cord decompression under endoscopic visualization with minimum trauma.

## Graphical abstract

The procedure and clinical results of anterior full‐endoscopic single‐port double transcorporeal spinal cord decompression were introduced. It can treat for the treatment of patients with noncontinuous two‐segment cervical spondylotic myelopathy with minimally invasive procedures, and meanwhile, reduce injury, reduce the risk of complications, and obtain a good clinical result.

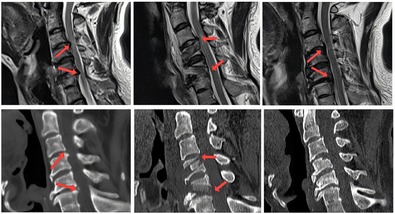

## Introduction

Noncontinuous cervical spondylotic myelopathy (CSM), also known as skip cervical spondylotic myelopathy, is a specific degenerative disease of the cervical spine that is relatively rare clinically and is characterized by varying degrees of degeneration of discontinuous cervical segments resulting in spinal cord compression, with the normal space often located in the middle of the diseased segment.^1^ Once diagnosed, early surgical treatment is needed with the aim of reducing spinal cord compression and saving and preserving the function of the spinal cord.^2^ Studies have shown that therapy within 6 months of symptom onset provides the best chance of recovery.^3^

Since the advent of anterior cervical decompression and fusion (ACDF) in the 1950s, it has gradually become an effective treatment for discontinuous CSM due to its high fusion rate and good clinical results.^4^, ^5^ The decompressive fusion of multiple segments is more prone to complications such as dysphagia, wound infection, and higher revision rates.^6^, ^7^ For noncontinuous multilevel cervical spondylotic myelopathy, when skip anterior cervical discectomy and fusion is used, the middle segment will bear more stress generated by the upper and lower fusion segments and accelerate the degeneration, which is faster than the upper and lower adjacent segments.^8^ Some have used long‐segment anterior fusion including normal middle segment (IS) for the treatment of discontinuous cervical spondylotic myelopathy, in order to reduce the stress on the fusion structure on IS and avoid adjacent segment degeneration (ASD) of IS. However, it sacrifices a large range of cervical motion, and there are more opportunities for anterior cervical tissue dissection and important organ tissue damage.^9^ In addition, various surgical techniques have been explored, including anterior cervical artificial disc replacement (ADR),^10^, ^11^ and hybrid surgery (HS).^12^, ^13^ Although these conventional decompression techniques have reported good spinal cord results, the optimal surgical approach and the best biomechanical choice remain controversial.^1^, ^11^, ^14^, ^15^, ^16^ The conventional open surgery described above is associated with many serious complications and significant medical trauma and surgical risks.

**FIGURE 1:**
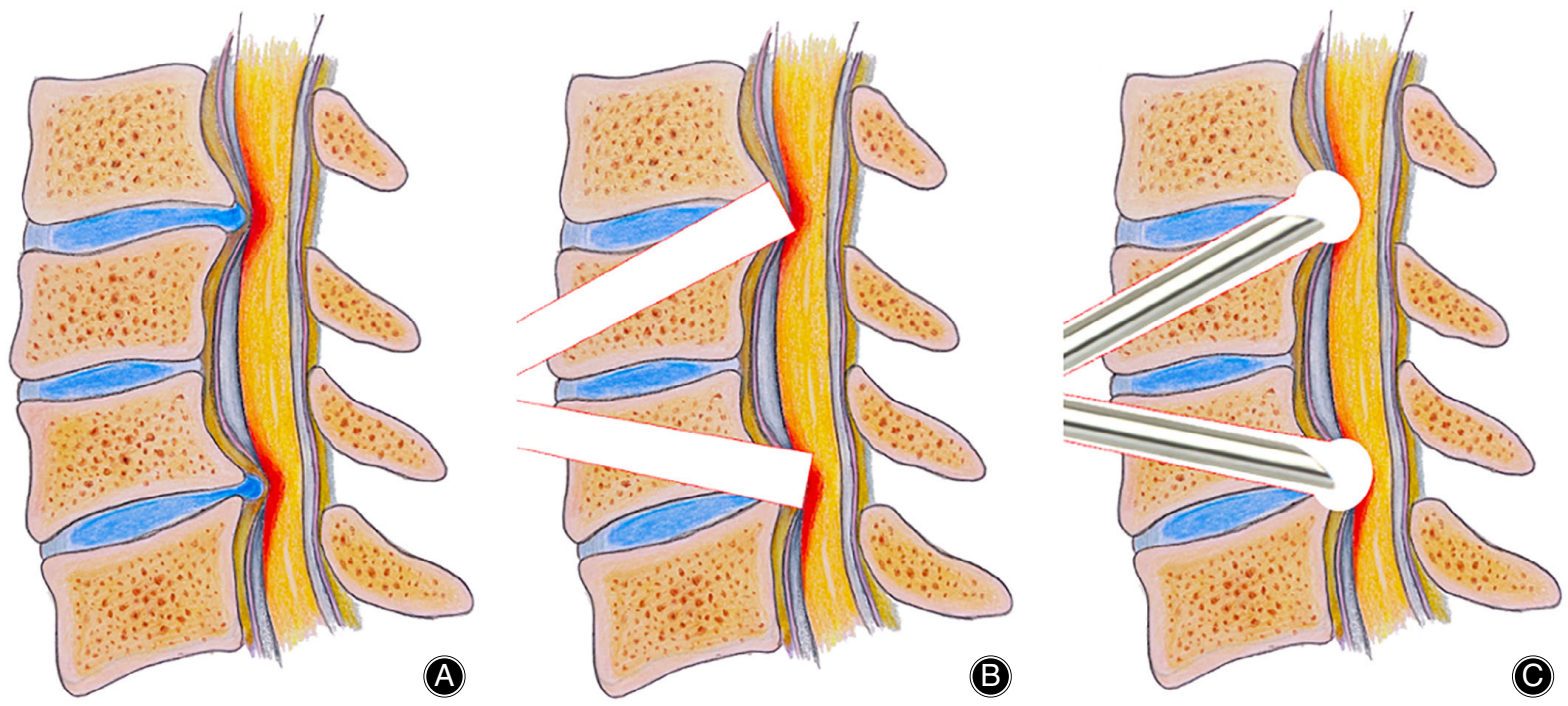
Noncontinuous two‐segment CSM (A). Two bone channels were established by the trephine through the vertebral body oblique upward and downward to the herniated disc osteophyte complex (B). The full‐endoscopic system could decompressive the spinal cord through the channels (C).

In order to overcome the many shortcomings of traditional open surgery, spinal surgery is constantly becoming minimally invasive.^17^ The coaxial full‐endoscopic technique using water as the medium has been gradually applied to treat various degenerative spinal diseases due to many advantages such as less trauma, clear vision, fewer complications, and faster postoperative recovery. Full endoscopic surgery offers a novel method for treating degenerative cervical spine conditions. Anterior surgery permits direct decompression and preserves the posterior cervical components' integrity. Depending on the technique, there are two primary types of anterior cervical endoscopic surgery: anterior endoscopic cervical discectomy (AECD) and anterior endoscopic cervical transcorporeal decompression (AECTcD).^18^ Anterior cervical endoscopy is mainly used for the treatment of single‐level cervical disc herniation or cervical spondylotic myelopathy, with limited indications.^19^ After reviewing the literature, to date, no studies have described the use of anterior total endoscopic techniques in cases of two‐segment spinal cervical spondylosis.^20^ In this study, we have the following objectives: (i) to propose a new technique, anterior full‐endoscopic single‐port double transcorporeal spinal cord decompression, and to present its technical features and details (Figure 1); and (ii) to report our short‐term clinical and radiographic results in order to assess its efficacy and safety in noncontinuous two‐segment CSM.

## Materials and Methods

### Patient Characteristics

**FIGURE 2:**
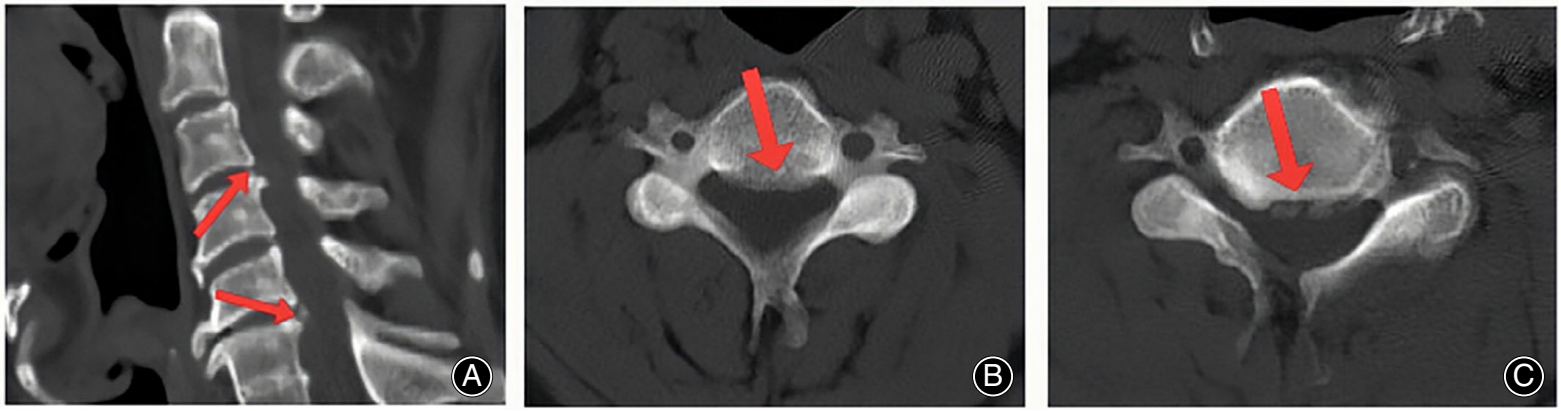
Preoperative CT scan. The sagittal section showed that the C3/4 and C5/6 level intervertebral disc‐osteophyte complex was formed (arrow) (A). The transverse section showed that the intervertebral disc‐osteophyte complex formed at the C3/4 level (arrow) (B) and C5/6 level (arrow) (C).

**FIGURE 3:**
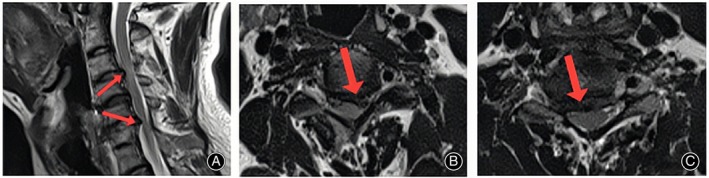
Preoperative T2‐weighted MRI scan. sagittal position showed that the spinal cord was obviously compressed by the C3/4 and C5/6 level intervertebral disc‐osteophyte complex (A), and the transverse position showed that the C3/4 level (B) and C5/6 level (C) intervertebral disc‐osteophyte complex compressed the ventral spinal cord.

This study was approved by the Ethics Committee (KLL‐2021‐319), and all patients had informed consent. Inclusion criteria were: (i) symptoms not improved after 3 months of standardized conservative treatment; (ii) clinical manifestations and signs of cervical spinal cord compression; (iii) the imaging examination revealed the patient's association in two noncontinuous segments, with a normal segment located in between; (iv) CT and MRI showed that the intervertebral disc‐osteophyte complex caused ventral compression of the spinal cord (Figures 2 and 3); and (v) the compression of the cervical spinal cord on imaging findings is consistent with clinical manifestations; Exclusion criteria were: (i) bony stenosis of the cervical intervertebral foramen and ossification of ligamentum flavum; (ii) previous history of cervical surgery; (iii) combined with tumor, infection, or intramedullary diseases; (iv) cervical instability; and (v) osteoporotic.

According to the above criteria, five patients with noncontinuous two‐level cervical spondylotic myelopathy underwent anterior percutaneous full‐endoscopic single‐port double‐vertebral bone‐access spinal cord decompression from February 2020 to May 2021. The characteristics of the patients were as follows (Table 1): There were two males and three females, with a mean age of 52.60 ± 4.93 (age range 48–60 years) and a mean disease duration of 24.40 ± 11.17 months (range of symptom duration: 12−40 weeks). Surgical intervention was performed on all patients at the C3/4 and C5/6 spinal levels.

**TABLE 1:** Characteristics of the patients.

Case	Gender	Age (years)	Surgery level	Duration of symptoms (weeks)	Surgical time (min)	Postoperative hospital stay (days)	Follow‐up time (months)
1	Male	48	C3/4−C5/6	12	180	3	33
2	Male	55	C3/4−C5/6	30	190	2	30
3	Female	60	C3/4−C5/6	40	200	2	27
4	Female	51	C3/4−C5/6	24	185	2	24
5	Female	49	C3/4−C5/6	16	170	1	24
	‐	52.60 ± 4.93		24.40 ± 11.17	185 ± 11.18	2.00 ± 0.71	27.60 ± 3.91

### Full‐Endoscopic Instruments

The spinal endoscopy system (SPINENDOS GmbH, Munich, Germany) included a 4.3 mm working channel, an outer sheath with a 6.9 mm diameter, a 30°‐angled scope with a continuous water irrigation system, and a trephine (Joimax, Karlsruhe, Germany) with an inner diameter of 6.5 mm and an outside diameter of 7.5 mm. The drill was made by NOUVAG AG (High‐speed burrs, Goldach, Switzerland).

### Operative Technique

#### Localization Puncture

Prior to the operation, gastric tubes were inserted and general anesthesia with endotracheal intubation was provided. The patient was placed in a supine position with suitable shoulder padding to maintain cervical hyperextension, and the shoulders and upper limbs were mildly tractioned to prevent them from obstructing the X‐ray fluoroscopic vision. The whole operation was performed under neurophysiological monitoring. Aa amount of 15 mL of iohexol was injected into the gastric tube to visualize and identify the location of the esophagus using C‐arm fluoroscopy. The C‐arm positioned the C3/4 vertebral segment and marked the body surface with a line. The two‐finger technique means utilizing the left index finger's middle finger to establish a safety gap through the manipulation of the visceral fascia sheath (VFS) and carotid sheath (CS) in opposing directions. Subsequently, a positioning needle is introduced, and the initial puncture point and needle direction are adjusted using C‐arm fluoroscopy.

#### Establish the Bone Channel

**FIGURE 4:**
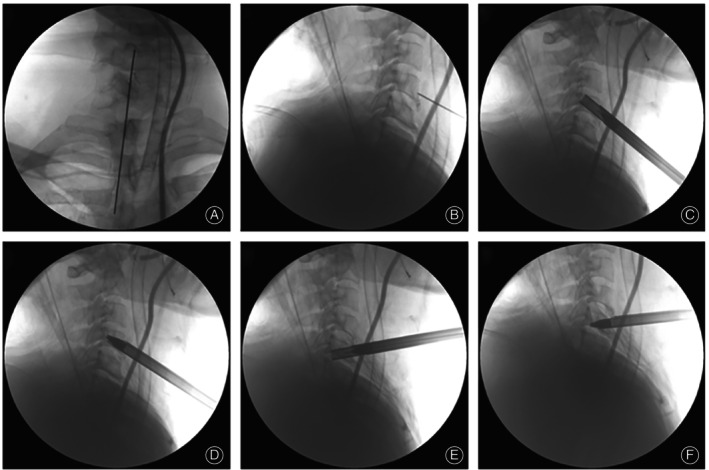
Intraoperative picture of C‐arm. The position of the positioning needle was determined through frontal and lateral perspectives (A, B). A trephine was screwed to establish a bone channel in the C4 vertebral body (C). The endoscope working sheath was inserted through the C4 vertebral body bone channel (D). The C5 vertebral body bone channel was used for the circular saw (E). The endoscope working sheath was arranged in the C5 vertebral body bone channel (F).

The starting puncture point near the lower edge of the C4 vertebral body, and then obliquely toward the superior posterior edge of the vertebral body (Figure 4A,B). A skin incision of approximately 8 mm in length was made at the center of the positioning needle, a three‐stage dilator and an operating trocar were placed along the positioning needle step by step, and the important tissues in the operating area were bluntly separated using the operating channel, the trephine was placed along the positioning needle, and the bone channel was established by rotary cutting with the trephine under the C‐arm observation, when the end of the trephine reached the target position, the bone strip was cut off by gently shaking the trephine. The full‐endoscopic system was then placed into the bone channel (Figure 4C,D).

#### Spinal Cord Decompression

**FIGURE 5:**
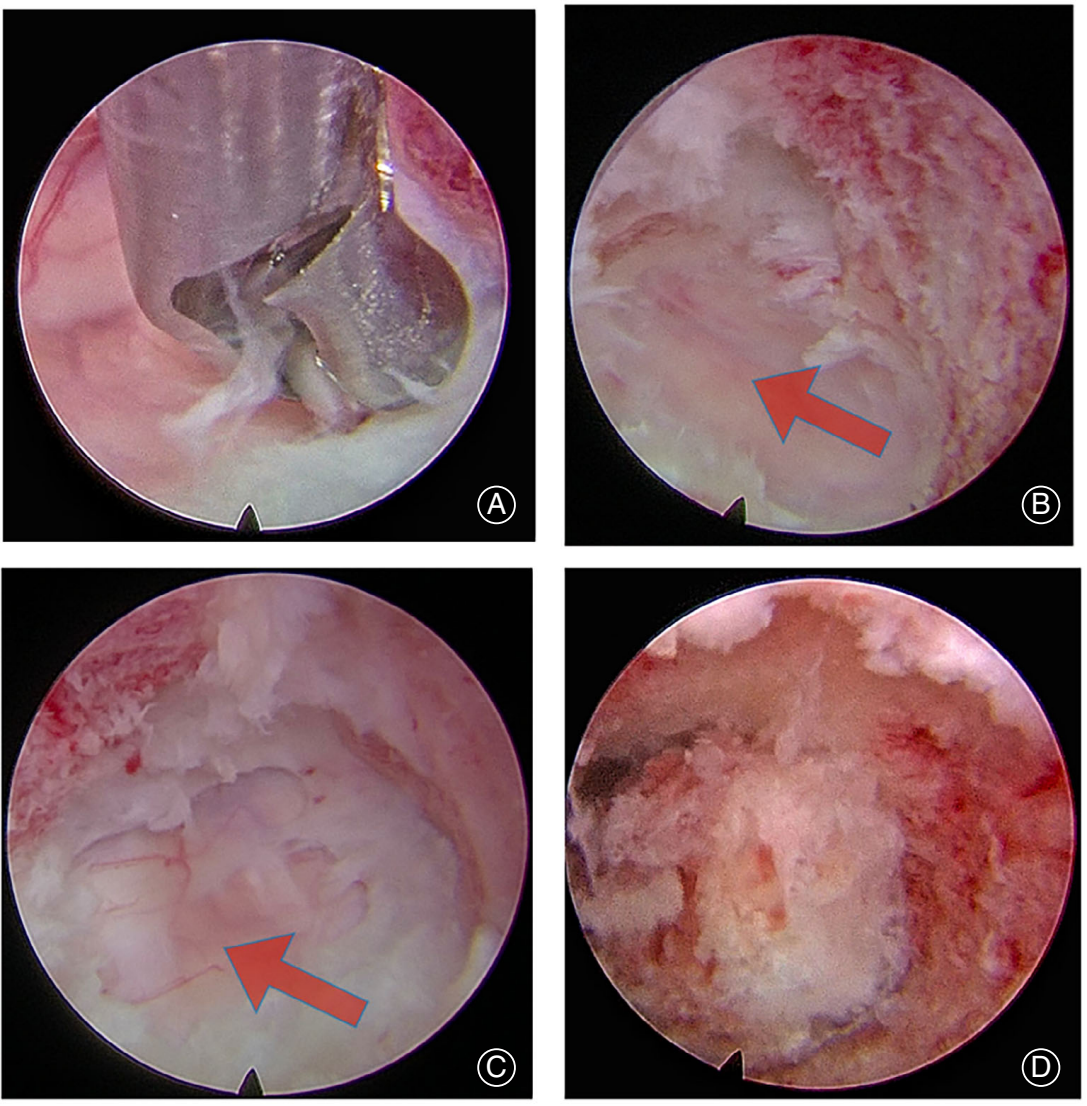
Endoscopic photographs. The herniated nucleus pulposus was removed with nucleus pulposus forceps. (A) The disc‐osteophyte complex was removed to complete the decompression, and C3/4 (B) and C5/6 (C) segment compressed dural sac was re‐expanded (arrow). Autogenous bone strips from the trephine are implanted into the bone channel (D).

Continuous saline irrigation was used to maintain a clear surgical field, endoscopic removal of residual tissue and vertebral bones in the bony canal was performed with rongeurs and a high‐speed diamond burr, then the herniated nucleus pulposus was removed with nucleus pulposus forceps to completely release spinal cord compression, with good dural pulsation observed endoscopically (Figure 5A). The vertebral canal and the bony channel were checked for active bleeding, and in the bony channel, autologous bone strips were implanted.

#### Management of the Other Lesion

Under endoscopic observation, the working trocar was advanced slowly to the anterosuperior margin of the C5 vertebral body, and a positioning needle was inserted along the working trocar. Under C‐arm observation, the position of the puncture starting point was adjusted, and the puncture approach needle was oriented obliquely toward the inferior posterior margin of the C5 vertebral body to establish a vertebral bony channel to the location of the lesion using circumferential rotational cutting, placing the endoscopic operating system in the bony channel (Figure 4E,F). In a similar fashion, the C5/6 lesion was resected to obtain precise and appropriate decompression of the target area, and the dura was seen to be distended and well‐pulsed (Figure 5B). Finally, we rechecked for active bleeding in the vertebral canal and bone channel, reimplanted the previously removed autologous bone strip into the C5 vertebral bone channel, and withdrew the endoscopic operating system. It was determined that the incision was free of active bleeding and no drainage was required to be placed, and the surgical incision was sutured and fixed with a sterile dressing wrap. The patient was closely observed for the occurrence of postoperative cervical hematoma formation and respiratory distress, and symptomatic treatments such as decongestion and nerve nutrition were routinely given. After awakening from anesthesia, eat and drink normally and observe for any related complications.

### Outcomes Evaluation

Surgical evaluation: observe whether there are esophageal and tracheal injuries, neurovascular injuries, postoperative hematoma, infection and other complications during the operation. The operation time and postoperative discharge time were recorded. Clinical evaluation: the visual analogue scale (VAS) scores of patients were documented prior to the surgical procedure, as well as at 1 week, 6 months, 12 months, and 24 months postoperation, in order to assess the level of pain experienced. Additionally, the Japanese Orthopaedic Association (mJOA) scores were recorded to evaluate the patient's neurological function. Radiographic evaluation: imaging examination was performed after operation and 24 months after operation to evaluate the curative effect. MRI scans were examined to assess the efficacy of intervertebral disc‐osteophyte complex removal and spinal cord decompression. CT scans were utilized to evaluate the condition of the bone passage. Furthermore, X‐rays of cervical hyperextension and flexion were reviewed during the final follow‐up to determine the presence of cervical spine instability.

### Statistical Analysis

The statistical analyses were conducted using the SPSS program. The mean ± standard deviation was used to represent the data. The paired *t*‐test was used to compare preoperative ratings with follow‐up scores at each time point. The value of *p* < 0.05 was considered statistically significant.

## Results

### General Results

All patients were successfully operated on by the same experienced endoscopic operator, and there were no complications such as esophageal and tracheal injury, nerve injury, vascular injury, and postoperative infection. In this study, one patient had neck swelling for a short time after operation, but it did not compress the trachea, blood vessels or nerves, and it decreased about 2–4 h after operation. The incision was only about 8 mm. The average operation time was 185.00 ± 11.18 min (170–200 min). The average postoperative hospital stay was 2.00 ± 0.71 days (1–3 days). Following the surgical procedure, all patients experienced alleviation of their pain symptoms. Over time, the intensity of pain steadily diminished, and no pain exacerbation was seen during the following period. During the course of the follow‐up period, there was a steady improvement observed in the symptoms associated with spinal cord compression, including numbness and paralysis of the limbs.

### Clinical Outcomes

**FIGURE 6:**
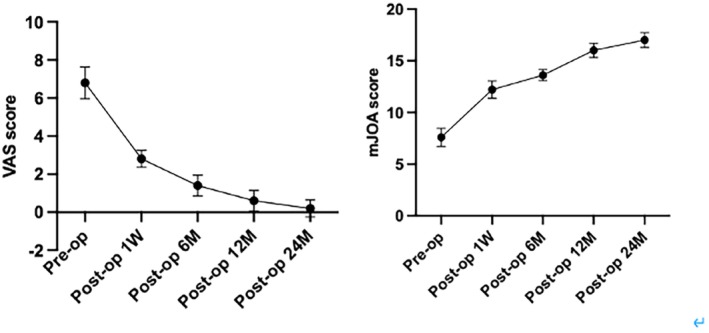
The change trend of VAS and mJOA scores at preoperative, postoperative, and follow‐up time points.

The VAS scores and mJOA scores of patients before the operation and at 1 week, 6, 12, and 24 months after the operation were as follows. There were significant differences between preoperative and postoperative time points (*p* < 0.05) (Tables 2 and 3). Figure 6 showed the trend of VAS and mJOA scores at preoperative, postoperative, and follow‐up time points.

**TABLE 2:** Preoperative and postoperative VAS scores.

Case	Preoperative	Postoperative 1 week	Postoperative 6 months	Postoperative 12 months	Postoperative 24 months
1	8	3	1	0	0
2	6	3	1	1	0
3	7	2	1	0	0
4	7	3	2	1	1
5	6	3	2	1	0
	6.80 ± 0.84	2.80 ± 0.45*	1.40 ± 0.55*	0.60 ± 0.55*	0.20 ± 0.45*

* *p* < 0.05 compared with preoperative values.

**TABLE 3:** Preoperative and postoperative mJOA scores.

Case	Preoperative	Postoperative 1 week	Postoperative 6 months	Postoperative 12 months	Postoperative 24 months
1	8	12	14	17	18
2	7	13	14	15	17
3	7	12	13	16	17
4	7	11	13	16	16
5	9	13	14	16	17
	7.60 ± 0.89	12.20 ± 0.84*	13.60 ± 0.55*	16.00 ± 0.71*	17.00 ± 0.71*

* *p* < 0.05 compared with preoperative values.

### Radiographic Outcomes

**FIGURE 7:**
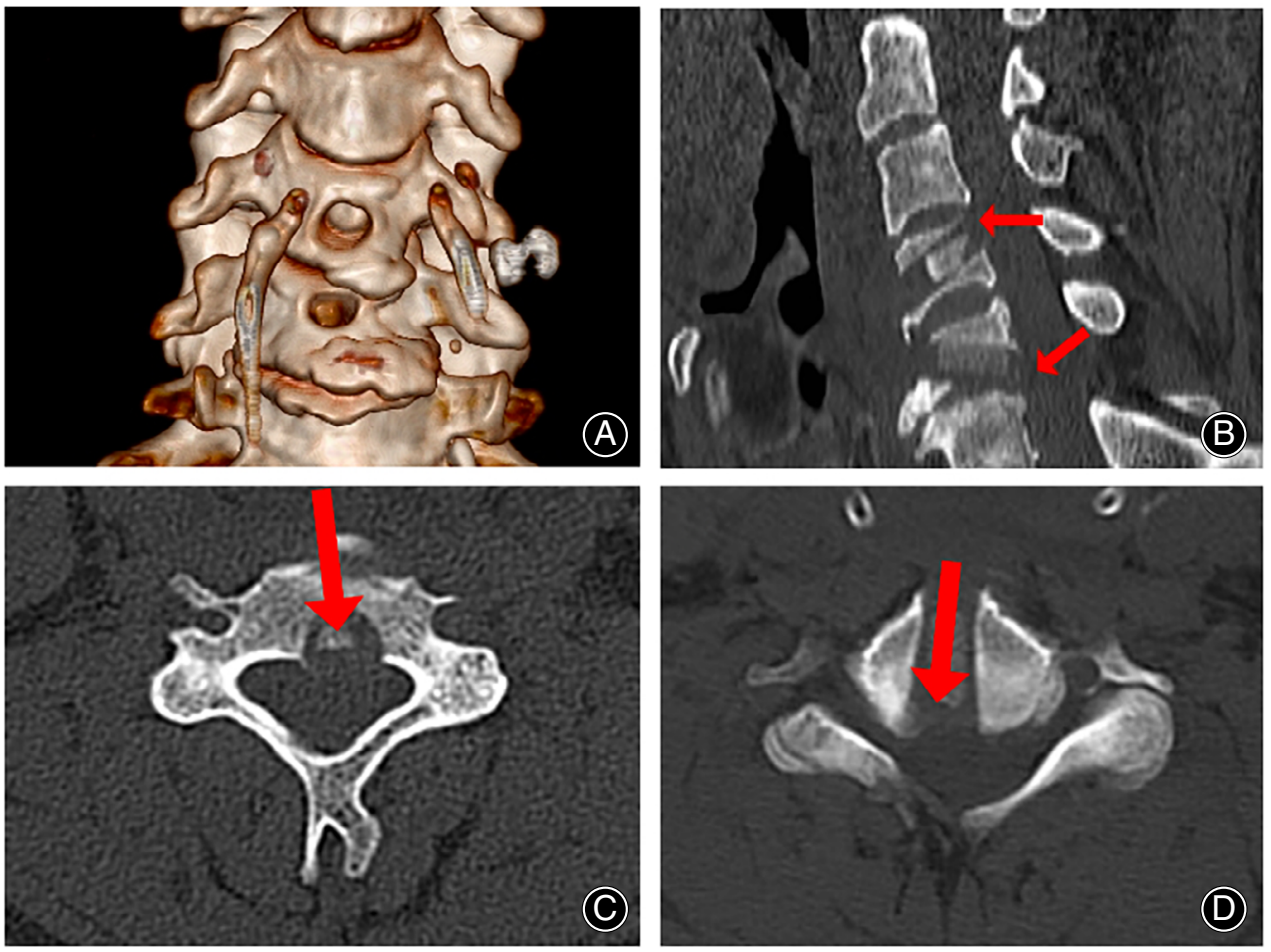
Postoperative CT image. Postoperative CT three‐dimensional reconstruction shows the entry location of the bone channel location (A). The sagittal position showed 3/4 and 5/6 cervical intervertebral disc‐osteophyte complex removed, showing the location and orientation of the channels (arrow) (B).The transverse view showed that the C4 and C5 vertebral bodies were complete in the channel, and the bone graft was implanted (arrow) (C, D).

**FIGURE 8:**
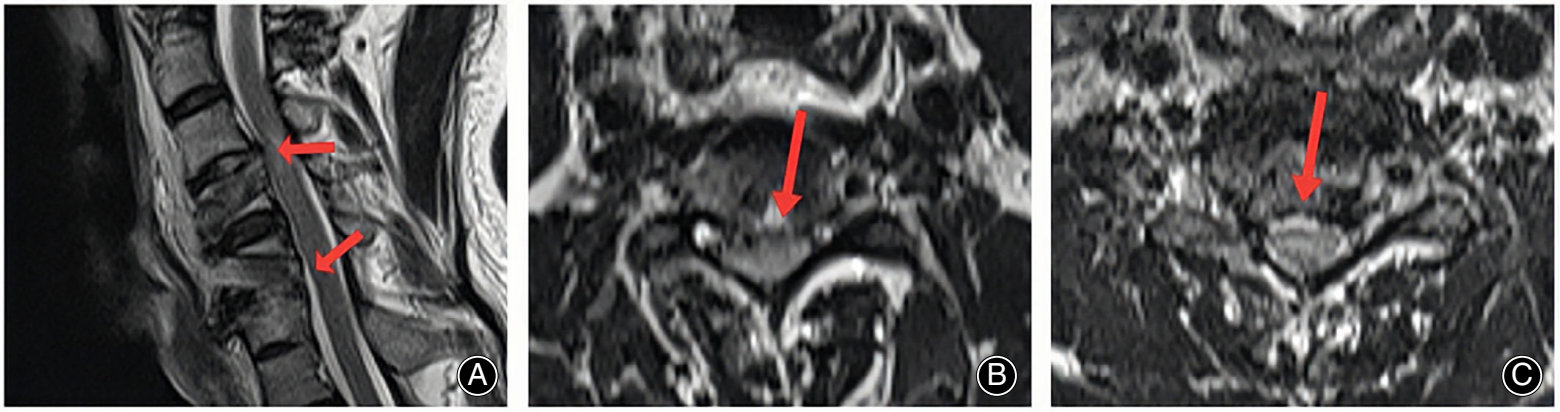
Postoperative T2‐weighted MRI images. The sagittal (A) and transverse images show adequate spinal cord decompression in C3/4 (B) and C5/6 (C).

**FIGURE 9:**
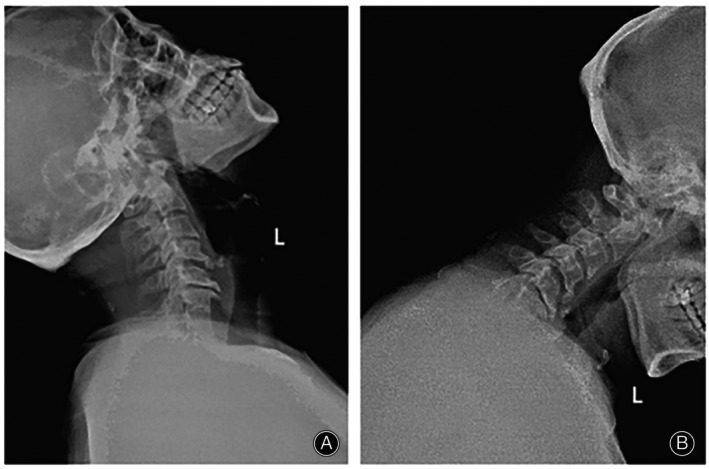
X‐ray at 24 months after the operation. The cervical spine dynamic radiograph showed that the physiological curvature of the cervical spine remained good without cervical instability (A, B).

**FIGURE 10:**
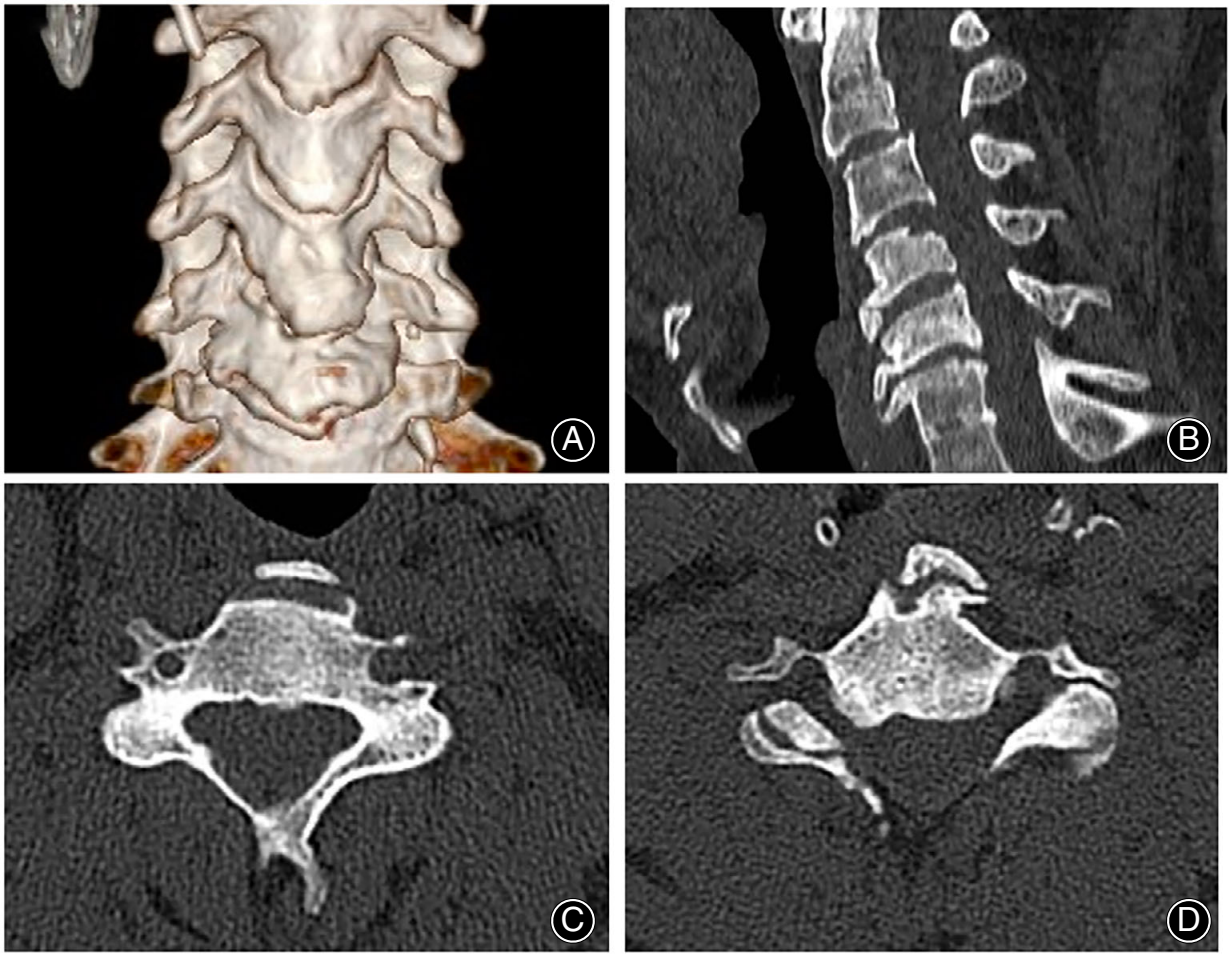
Follow‐up CT images. The bone channel almost disappeared 24 months after surgery, including the three‐dimensional view (A), sagittal reconstruction view (B), and axial plane view (C, D).

**FIGURE 11:**
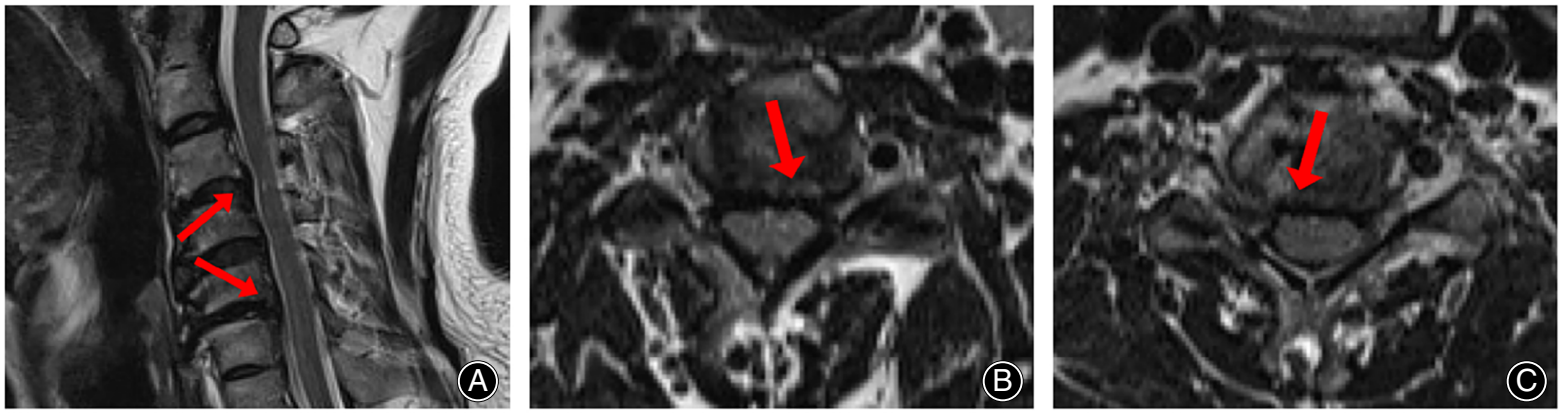
Follow‐up T2‐weighted MRI images. Sagittal (A) and transverse views (B, C) showed that the spinal cord of 3/4 and 5/6 of the neck was not compressed, and the decompression was adequate (arrow).

Postoperative CT (Figure 7) and MRI (Figure 8) scans showed adequate resection of the intervertebral disc‐osteophyte complex at the C3‐4 and C5‐6 levels, complete decompression of the spinal cord, and intact placement of the autogenous bone strips implanted in the channel. Cervical hyperextension and hyperflexion radiographs were taken at the 24‐month postoperative follow‐up, and no cervical kyphosis deformity or cervical instability was seen to occur (Figure 9). CT scan and three‐dimensional reconstruction of the cervical spine showed complete healing of the vertebral bone channel. No obvious vertebral fracture or collapse of the bony channel was observed (Figure 10). MRI of the cervical spine showed adequate spinal cord decompression without recurrent compression (Figure 11).

## Discussion

Due to the presence of a normal disc tissue in the middle of the degenerated segment in noncontinuous two‐segment CSM, the choice of surgical approach for this condition remains controversial. In this study, we showed that anterior full‐endoscopic single‐port double transcorporeal spinal cord decompression is an effective and safe surgical approach for noncontinuous two‐segment CSM. At 2‐year follow‐up, neurologic function was significantly restored, and both VAS and mJOA scores improved progressively. The intervertebral disc‐osteophyte complex was removed through the vertebral bone channel, the spinal cord was completely decompressed, and all patients achieved a well‐healed bone channel with no recurrence of symptoms.

### Application of Anterior Cervical Endoscopy

Endoscopic transforaminal discectomy for cervical disc herniation has been widely utilized in clinical practice; however, it has a number of drawbacks. Low disc height and anterior osteophytes in the operative segment will limit the use of the transdiscal approach and cause irreversible disc damage as a result of intraoperative instrumentation through the disc, resulting in decreased intervertebral space (IVS) and accelerated degeneration of the disc tissue.^21^, ^22^ To reduce iatrogenic injury to the disc, maintain cervical spine stability, and preserve cervical motion segments, some studies have attempted anterior endoscopic cervical transcorporeal decompression (AECTcD), after which the vertebral body bone channel usually heals spontaneously.^23^, ^24^ The surgical indications for anterior cervical endoscopic surgery are limited, consisting primarily of single soft herniated disc tissues or single stenotic lesions, and do not reveal an effective decompression impact on spinal cord compression symptoms induced by the disc‐osteophyte complex. Patients with cervical spondylosis of the spinal cord are often excluded from case selection or deemed surgical contraindications. Quillo‐Olvera *et al*. reported three successful cases of anterior endoscopic transcorporeal spinal decompression guided by CT‐based intraoperative spinal navigation for single‐segment CSM, and all patients had effective cervical decompression, exhibiting improvement in preoperative symptoms and the absence of procedure‐related problems.^25^ Kong *et al*.^19^ utilized a complete endoscopic procedure with a transcorporeal approach to examine 32 patients with single‐segment CSM at a 2‐year follow‐up. The results suggested that this approach for patients with single‐segment CSM offered several benefits, including reduced damage while avoiding the complications of ACDF or ACDR, favorable short‐term clinical outcomes, and no significant effect on cervical stability. The selection of patients for anterior percutaneous total endoscopy is now restricted to single‐segment CSM or CDH lesions, and no investigations have been conducted on lesions involving two or more segments. In this case, spinal cord injury was caused by a herniated disc osteophyte complex that compressed the ventral spinal cord. Some writers consider that anterior surgery is more appropriate if the compression is ventral since it offers immediate decompression.^2^, ^26^ Posterior surgery is harder to alleviate the central CSM ventral to the compressor and has a higher risk of spinal cord damage, making achieving the desired clinical result more challenging.^27^ Based on these factors, the anterior method is the superior option for this situation. In conclusion, this operation is mainly suitable for the lesion of noncontinuous double‐segment spinal cord compression caused by the ventral spinal cord compression factors such as the disc‐osteophyte complex.

### Complications of Cervical Endoscopy

Complications of cervical endoscopy vary depending on the choice of access and commonly include transient neurologic deficits, dural tears, visceral injuries, dysphagia, hematoma, and hoarseness.^28^, ^29^, ^30^, ^31^ In anterior cervical endoscopy, dysphagia is the most reported, while visceral injuries occur mainly in the esophagus.^32^, ^33^ For anterior endoscopic cervical transcorporeal decompression (AECTcD), some of the literature has reported the possibility of endoscopic flushing fluid flowing down the fascial space into the peripheral space and mediastinum leading to neck swelling for a short period of time after the procedure.^19^, ^24^ In addition, as a transcorporeal nonfusion technique, the potential complications of bone channel collapse or vertebral fracture and recurrence of disc herniation need to be noted.

### Key Points of Technology

#### Safe Puncture Positioning

Safe puncture positioning is one of the keys to this technique. The iohexol contrast in the inserted gastric tube demonstrates the esophageal alignment under C‐arm fluoroscopy, allowing the puncture needle to be placed with minimal risk of injury to the esophagus and trachea. Using the “two‐finger” technique typically used in anterior cervical minimally invasive surgery, in which the surgeon uses the index and middle fingers of the left hand together to insert longitudinally along the interstitial space between the carotid sheath and the trachea and esophagus and to open the two fingers during the process of pressing down from shallow to deep. The middle finger pushes the visceral fascial sheath surrounding the trachea and esophagus to the medial side, and the index finger pushes the carotid sheath surrounding the large blood vessels of the neck to the lateral side to expand the gap.^34^ When the fingertip touches the front of the vertebral body or the intervertebral disc, the downward pressure operation is completed to form a safe puncture area without damaging important tissue structures. Through this safe puncture area, the positioning needle enters and reaches the surface of the intervertebral disc or vertebral body. This patient's lesion segments are located at C3/4 and C5/6. After completing the decompression of one segment, the previous steps can be repeated using the two‐finger method to find another segment again to make a skin incision and establish access. However, repositioning the puncture will have the following shortcomings: (i) significantly extending the duration of the operation; (ii) possibly causing bleeding in the incision or bone channel by repeatedly pushing the anterior cervical tissues with the two‐finger approach; and (iii) affecting the aesthetics, increasing the number of intraoperative radiographs and increasing the risk of injury to the anterior cervical vital organs and tissues. We ingeniously adopted the obliquely upward direction of the bony channel of the C4 vertebral body and the obliquely downward direction of the C5 vertebral body based on the arguments above. When a lesion is treated, the working sleeve is not completely removed but only withdrawn to the entrance of the vertebral body. The working sleeve is then used to bluntly separate a portion of the tissue in the endoscopic view and to move down a short distance to the other target vertebral body, where a puncture needle is inserted for positioning and establishing the bone passage. This technique permits the creation of a dual vertebral bone channel with a single skin incision for spinal cord decompression, therefore considerably lowering the risk of harm to essential anterior cervical structures. This section must be aware of anterior cervical vascular nerve injury and requires a comprehensive understanding of anatomy, meticulous operative technique, and demonstrated experience with cervical endoscopy.

#### Precise Establishment of Bone Channel

Establishing a precise bone channel is also essential. A too‐deep channel can cause spinal cord damage, and a deviated channel can result in surgical failure. Preoperative CT and MRI aid in determining the precise anatomical site of the lesion, while intraoperative C‐arm real‐time monitoring and fine‐tuning of the working channel orientation during drilling aid in ventral decompression. Previous studies have pointed out that the bony channel drilling starting position in the coronal plane should be close to the center of the anterior edge of the vertebral body to reduce the risk of Horner syndrome.^35^, ^36^ In contrast, the direction of the bony channel in the cross‐sectional plane is adjusted to the left and right according to the location of the disc protrusion. The direction in the sagittal plane is oblique from the upper edge of the lower endplate to the disc protrusion to reduce the impact on the spine's stability. Some researchers and others have proposed using the O‐arm in anterior transcorporeal minimally invasive surgery as a means of surgical planning and intraoperative targeting to correct the surgical path and assess proper instrument placement.^25^, ^37^, ^38^ Chu *et al*.^39^ drilled holes in the vertebral body using a high‐speed grinding drill guided by mixing methylene blue bone wax to target the herniated nucleus pulposus; however, the channel direction was frequently erratic. Du *et al*.^40^ utilized a trephine saw to produce a smooth and intact bone channel in the cervical vertebral body and permitted the extraction of autologous bone as an implant to restore the bone channel. In this investigation, the trephine was used to create the bone channel. The intercepted bone strips were effectively cut and grafted into the channel to lessen the risk of rejection and channel collapse, hence expediting the bone channel's healing process. In the second year of follow‐up, the fracture repair was in the reconstruction period, osteoclasts and osteoblasts were coupled circularly, the bone marrow cavity was reconstructed, the callus gradually disappeared, the bone passage was basically healed, and the vertebral body recovered to its previous morphological and biomechanical level.^41^ A preoperative bone density assessment was also recommended to minimize an increased risk of postoperative vertebral fracture owing to osteoporosis.

#### Precaution of Endoscopic Operation

The endoscope's enlarged, clear surgical image enables the surgeon to precisely discriminate between the nerve and surrounding tissues and undertake delicate, painstaking procedures. Usually, the spinal cord decompression process of endoscopic surgery has less bleeding, but the accumulation of blood in the case of poor hemostasis will affect the clarity of endoscopic surgical view and increase the risk of operation. Continuous irrigation with aqueous media can reduce intraoperative bleeding, appropriately raise the saline suspension height, and increase hydrostatic pressure to control bleeding to maintain a clear endoscopic view. Venous bleeding can be blocked by small bone fragments created by the drill and the pressure of the irrigating fluid. In addition, extra care should be taken when decompressing the dural sac periphery, which is rich in venous plexus and causes bleeding that has a greater impact on the field of view, and low energy radiofrequency spot hemostasis can be used. Complete spinal cord decompression requires the removal of the disc‐osteophyte complex utilizing a nucleus pulposus forceps and a high‐speed grinding drill. Endoscopic use of a blunt hook probe to assess if osteophytes at the posterior margin of the vertebral body have been removed; indications of spinal cord pulsation indicate successful dural decompression.^23^, ^36^

### Limitations and Strengths

The following limitations of this study should be noted: (i) the operation is mainly for patients with ventral spinal cord compression, and patients with dorsal compression factors such as ossification of the ligamentum flavum are not suitable for this operation; (ii) the steep learning curve is the main obstacle that limits the promotion of this surgery. Continuous improvement of surgical techniques and improvement of safety are the future research directions. This technique achieves direct and precise decompression of noncontiguous two‐segment spinal cord segments with maximum preservation of cervical segment motion after decompression; and (iii) due to the relatively small number of clinical cases that meet the surgical indications for noncontiguous double‐segment and strict selection criteria, the included cases were limited, and the follow‐up time was short, which may lead to potential bias in assessing the efficacy of this new technique.

This study has several strengths as well: (i) for non‐contiguous double‐segment CSM, unlike traditional long‐segment fusion internal fixation surgery, this study proposes a new non‐fusion endoscopic surgical technique that maximizes the preservation of cervical motion segments after decompression; (ii) it is capable of precisely and accurately targeting and removing non‐contiguous double‐segment lesions within a short surgical time; (iii) furthermore, this method minimizes damage to the anterior cervical tissue structures, reducing risks; and (iv) The use of a single skin incision for the procedure contributes to a more aesthetically pleasing outcome.

## Conclusions

As the first report of treating noncontinuous two‐segment CSM using a full endoscopic technique, its outcome was satisfactory based on the clinical and radiological evaluation. This technique achieves direct and precise decompression of noncontiguous two‐segment spinal cord segments with maximum preservation of cervical segment motion after decompression. It shows many advantages, such as small incision, minimal trauma, rapid postoperative recovery, and no need for internal fusion fixation.

## Conflict of Interest Statement

The authors declare that there is no conflict of competing financial or non‐financial interests.

## Ethics Statement

This study was approved by the Ethics Committee of The Affiliated Hospital of Zunyi Medical University (KLL‐2021‐319). All patients signed informed consent forms. All methods were performed in accordance with the relevant guidelines and regulations.

## Author Contributions

All authors had full access to the data in the study and take responsibility for the integrity of the data and the accuracy of the data analysis. Conceptualization: WBL and ZJX; methodology: WBL and WJK; investigation: GC and XYL; resources: WBL, FJW, and YQ, writing—original draft: GC and XY; writing—review and editing: WBL and ZJX; visualization: WJK; and supervision: ZJX and WBL.
